# Application of Implantable Polylactic-Co-Glycolic Acid Microcapsule in Repairing Alveolar Bone Defects

**DOI:** 10.1155/2021/5580785

**Published:** 2021-07-27

**Authors:** Jun Jiang, Jianpeng Xiao, Dongqing Wang, Huazhong Cai

**Affiliations:** ^1^School of Pharmacy, Jiangsu University, 301# Xuefu Road, Zhenjiang 212013, Jiangsu Province, China; ^2^Affiliated Hospital of Jiangsu University, Department of Medical Imaging, Zhenjiang 212001, Jiangsu Province, China; ^3^Affiliated Hospital of Jiangsu University, Department of Emergency, Zhenjiang 212001, Jiangsu Province, China

## Abstract

Alveolar bone defects (ABDs) were a perennial problem, especially in the aged. Bisphosphonates, especially etidronate sodium (ET), were frequently used in clinical treatment of ABD. However, the oral administration of ET had poor absorption (<1%). Therefore, optimization of a suitable dosage form substituted with ET to locally repair the ABD was a straightforward approach. Polylactide-co-glycolide (PLGA) is a biodegradable material and had been used in locally implanted medical devices. Therefore, an ET-PLGA microcapsule may help local delivery and prolong the activity of healing ABD. In this paper, a preparation method of ET-PLGA microcapsule was optimized by the single-factor investigation and response surface method. Subsequently, the rat ABD model was used to evaluate the enhancement effect of these microcapsules. Finally, the optimum parameters were determined as follows: 40% dichloromethane, 160 mg/mL PLGA, 10% internal aqua/oil phase, 4% PVA, and emulsifying for 10 min. These microcapsules were spherical in shape and fairly monodisperse in a particle size of 27,51 *μ*m (PDI = 0.3), encapsulation rate 96.6%, and drug loading 4.58%. Compared with the ET groups, the total healing volume of ABD in ET-PLGA groups was significantly increased (*P* < 0.05). ET-PLGA microcapsules significantly enhanced the effect of ET on ABD. This study provided important technical support for the treatment of ABD with bisphosphonates by local administration. This paper has an exploratory significance for the development of water-soluble bioactive components with low bioavailability for ABD.

## 1. Introduction

Alveolar bone defects (ABDs) continue to be a perennial problem, especially in the aged [1]. ABD is mainly caused by trauma, infection, periodontal disease, or congenital alveolar fenestration, which affected the mastication ability and quality of life [2, 3]. Bisphosphonates, antibiotics, or anti-inflammatory compounds have been wildly used to cure the ABD, but their ability to effectively achieve alveolar regeneration remains elusive [4, 5], especially the bisphosphonates.

In clinical treatment, bisphosphonates are the most commonly prescribed for antiresorptive drugs [6, 7]; for example, etidronate sodium (ET) was frequently chosen to increase bone mineral density and reduce the risk of fracture and was well tolerated [8, 9] by oral administration. However, its excretion levels by the renal system reached 38% to 73% in 24 h [10]. Furthermore, the poor absorption from the gastrointestinal tract was its major disadvantage, generally less than 1% [11]. Therefore, optimization of synthetic bone substitutes with ET to locally adjust the imbalance in bone remodeling seemed a straightforward approach to aid bone regeneration in ABD.

Among the available synthetic bone substitutes, polylactide-co-glycolide (PLGA) is an FDA-approved biodegradable material and had been used in locally implanted medical devices, including scaffolds [12–15]. PLGA composites could maintain the structural integrity of in situ placement, provide micro-/macropore space, and stabilize the bioadhesion of clots and quick biodegradation for rapid clearance [16]. PLGA microcapsule is an effective way to locally control drug release and can be easily adapted to complex defects in a less-invasive manner compared to conventional surgery [17]. Compared to the conventional drug delivery vector, PLGA also had been extensively applied to other delivery systems such as nanocrystals and microspheres [18–20].

According to the internal structure, PLGA microparticles are classified into microspheres and microcapsules [21]. In microcapsules, vesicular particles consist of a polymer shell surrounding a single core (mononuclear) or multicores which mainly appropriate for encapsulating hydrophilic drugs. PLGA implants gradually released encapsulated drug in situ as they were degradated and prolonged the drug bioactivity through control degradation time [22, 23]. Theoretically, PLGA should be able to encapsulate water-soluble ET and increase its bioavailability and tissue repair by controlling release.

ABD repair generally lasts several months, so it is necessary to prolong the drug efficacy in the treatment of this disease [24]. An ET-loaded PLGA may help local delivery and prolong the activity of ABD healing in rats for a long period. However, there have been few reports on the fabrication of this delivery system. In this paper, a preparation method of Water_1_ (W_1_)/O (Oil)/W_2_ ET-PLGA microcapsule was optimized by the single-factor investigation and response surface method. Subsequently, the rats' ABD model was used to evaluate the enhancement effect of these microcapsules.

## 2. Materials and Methods

### 2.1. Ethics Statement

All animal experiments strictly comply with the Guidelines for Animal Experimentation of Jiangsu University (Zhenjiang, China), and the protocol was approved by the Animal Ethics Committee of this institution.

### 2.2. Materials and Chemicals

Etidronate sodium (purity > 99.8%) was purchased from Jizhi Biochemical Technology Co., Ltd. (Shanghai, China). PLGA (75/25) was purchased from Jinan Daigang Bioengineering Co., Ltd. (Jinan, Shanghai, China). The chromatographic distilled water was made in our laboratory. Chloral hydrate, polyvinyl alcohol (PVA), dichloromethane (DCM), ethyl acetate (EAC), and KOH were purchased from Titan Technology Co., Ltd. (Shanghai, China).

### 2.3. Preparation of ET-PLGA Microcapsules

**Figure 1:**
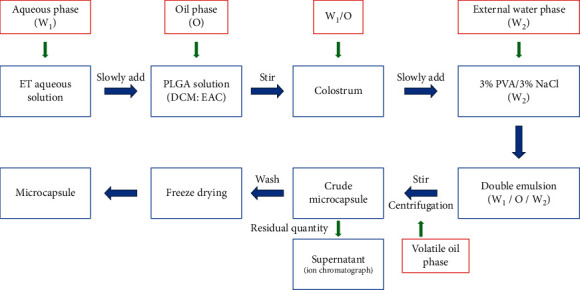
Preparation flow chart of ET-PLGA microcapsules.

The ET-PLGA microcapsules were prepared by improved double emulsion solvent—an evaporation method to form W_1_/O/W_2_ complex emulsion. The customized preparation parameters were set as follows: 40% DCM (DCM: EAC), 160 mg/mL PLGA, 10% internal aqua/oil phase, 4% PVA, and emulsifying for 10 min. The operation flow is shown in Figure 1.

### 2.4. Quality Evaluation of Microcapsules

The encapsulation efficiency (EE) of microcapsules was chosen as the main index to evaluate microcapsules, and the drug loading yield (DL) and particle size distribution (PSD) were also considered. EE (%) = [(amount of drug used in the formulation−Amount of residue in the supernatant)/Amount of drug used in the formulation] × 100%. Actual drug loading = (Total amount of drugs in microcapsules/Microcapsule weight) × 100%.

### 2.5. Determination of Drug Content

We accurately weighed etidronate sodium, prepared 5 mg/mL standard solution, diluted to 10 *μ*g/mL, 40 *μ*g/mL, 80 *μ*g/mL, 160 *μ*g/mL, 320 *μ*g/mL, and 400 *μ*g/mL standard solution. An Ics 600 ion chromatograph (Thermo Fisher Scientific Co., Ltd., Shanghai, China) was used to detect the peak area as the ordinate and the concentration as the abscissa, and linear regression was used to obtain the standard curve equation. In order to determine the residual amount of ET in the supernatant as described in Figure 1, 5 mL of the supernatant of ET-PLGA microcapsule preparation was obtained, successively filtrated by using a C_18_ SPE column and 0.22 *μ*m aqueous membrane, and then, injected into an ion chromatograph for analysis.

### 2.6. Single-Factor Experiment

The entrapment rate as the main index and the main factors affecting the formation of microcapsules, such as oil phase composition (DCM: EAC), PLGA concentration, volume ratio of internal water phase to oil phase (RWO), emulsification time, and PVA concentration, were investigated. During the single-factor investigation, the encapsulation rate and particle size were measured as evaluation indicators.

### 2.7. Response Surface Experiment

Based on single-factor experiment, response surface experiment was designed by selecting 3 factors (Proportion of DCM, PLGA concentration and RWO) which have an obvious influence on the quality of microcapsules. The response surface was designed by Box–Behnken, and the encapsulation rate was taken as the index.

### 2.8. Animals and Administration

Thirty SD rats (220 ± 20 g) were supplied by the Laboratory Animal Center of Jiangsu University. Before the experiment, all rats were given 14 days of adaptation period with a standard laboratory rodent diet (calcium content 0.5%) and tap water under climate-controlled conditions (55% humidity, 25°C, and 12 hours alternating day and night). After the accommodation period, the rats were anesthetized with 10% urethane (10 mL/kg) by intraperitoneal injection. After fixation, the alveolar bone of rat was exposed and a round hole bone defect was made by using a dental drill (*T*_3_, Sirona Dental Systems Co., Ltd., Shanghai, China) near the first molar. In order to prevent the high temperature of the operation area, the rats' ABD was made by intermittent and low-speed drilling. After establishing the ABD model, the ET powder or prepared ET-PLGA microcapsule were implanted into the bone defect immediately, and the gingiva was sutured carefully. Rats were divided into 6 groups (*n* = 5); they were the blank control group (CON), ABD model group (ABD), ET low-dose group (ET-L, 2 mg), ET high-dose group (ET-H, 10 mg), ET-PLGA low-dose group (ET-PLGA-L, 2 mg), and ET-PLGA high-dose group (ET-PLGA-L-H, 10 mg). In the CON group, no diaphragm was implanted and the wound was sutured directly. All the experimental protocols were approved by the Animal Ethics Committee of Jiangsu University.

### 2.9. Collection and Analysis of Alveolar Bone Samples

Within one month after the operation, cone-beam computed tomography (CB-CT, kawa i-cat 17–19, Imaging science international LLC, USA) was used to scan the ABD of rats every week. The main parameters of CB-CT were as follows: reconstruction solvent size was 8 cm ╳ 8 cm (diameter ╳ height), resolution was 125 pixels, exposure was mAs = 37.07, KVP = 120, and acquisition time = 26.9 seconds. Mimics Research 20.0 software was applied to analyze the CT image and calculate defect volume.

### 2.10. Statistical Analysis of Data

Statistical analyses were conducted by one-way ANOVA followed by Tukey's test (GraphPad Prism 5.0) for comparing all groups. Data were presented as mean value ± SD. The *P* value < 0.05 was considered as statistically significant.

## 3. Results

### 3.1. Methodological Validation

**Figure 2:**
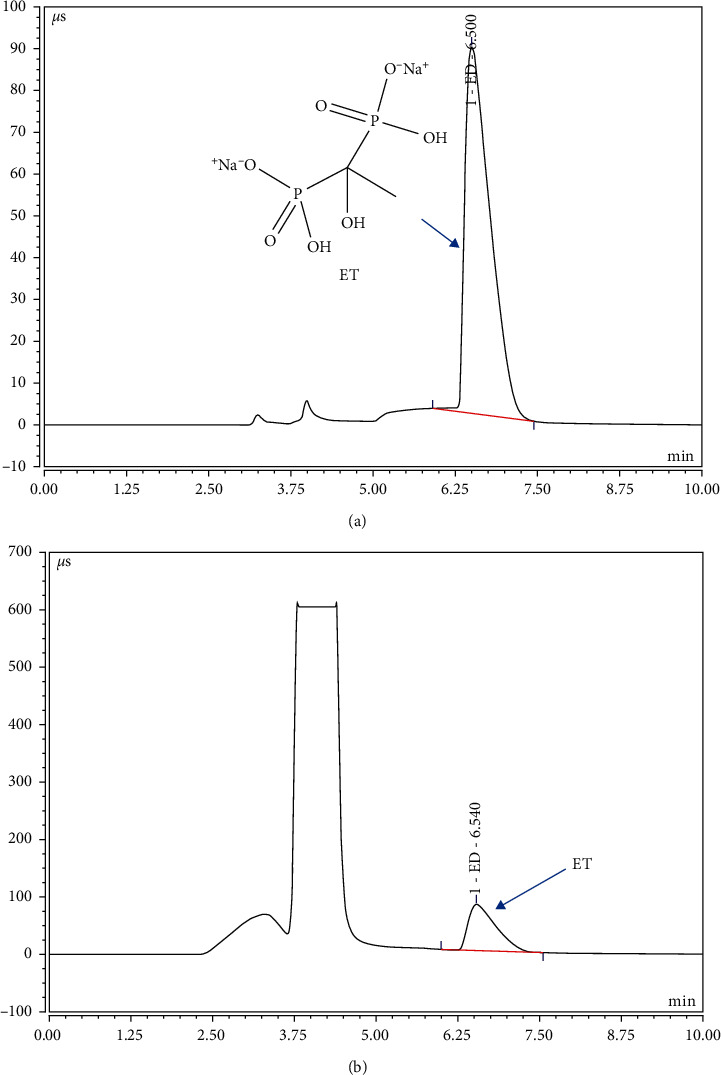
Determination of sodium etidronate by ion chromatography. (a) Chromatogram of the ET standard solution; (b) chromatogram of ET detection in samples.

Ion chromatography was established for the determination of ET residue in the supernatant by an external standard method (Figure 2). The mobile phase was 30 mmol/L KOH solution with isocratic elution. The regression equation was obtained by linear regression of concentration *Y* with peak area *X*. The standard curve was *Y* = 0.251, *X*−0.184, *R*^2^ = 0.9996. The results showed that there is a good linear relationship between 10 *μ*g/mL and 400 *μ*g/mL. The 3 or 10 times of the relative standard deviation of the analytical blank values was calculated as the limit of detection (LOD) and limit of quantitation (LOQ). The LOD and LOQ were 0.6 ng and 2.1 ng, respectively. One sampling solution was treated according to Section 2.5 and then injected into an ion chromatograph for 6 times continuously to record the peak area of ET. The RSD was 2.34% which indicated that the precision is fine (Table S1). Similarly, when the sample solution was injected at 0, 2, 4, 8, 16, and 24 h, the RSD of the peak area of ET was 0.27%, indicating that ET was stable within 24 h under room temperature (Table S2). For repeatability, six samples with the same concentration were determined and the RSD of their contents was 0.37% (Table S3), indicating a good repeatability. The sample with known concentration was chosen, and, respectively, added into ET according to 1 : 1, 1 : 2, and 1 : 4 times, and the recovery rate was calculated ([Measured quantity − Original quantity] × 100/Amount added, %). The recovery was 99.6% with the RSD of 1.81% (Table S4). All these results showed that the ion chromatography method established in this paper can be well applied to optimize the preparation of ET-PLGA microcapsules.

### 3.2. Single-Factor Results

The oil phases (30%, 45%, 60%, and 75% dichloromethane, Table 1), PLGA concentrations (80, 120, 160, and 200 mg/mL, Table 2), PVA concentrations (1%, 2%, 3%, and 4%, Table 3), emulsification times (5, 10, 15, and 20 min, Table 4), and RWO (v/v 5%, 10%, 15%, and 20%, Table 5) were investigated by EE (%) and particle size (nm). The single factor results indicated that the best encapsulation efficiency was obtained when the proportion of DCM, PLGA, PVA, emulsification time, and RWO was 30%, 200 mg/mL, 4%, 10 min, and 5%, respectively.

### 3.3. Response Surface Test Results

**Figure 3:**
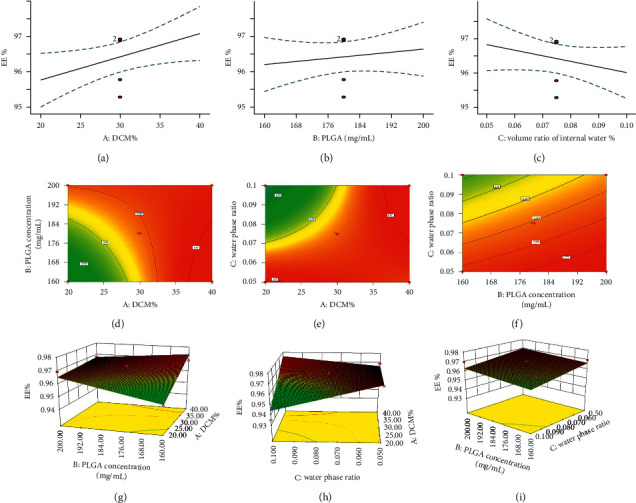
Response surface optimization results. (a) The effect of DCM ratio on EE%; (b) the effect of PLGA concentration on EE%; (c) the influence of the ratio of internal water phase to EE%; (d) 2D panel of PLGA-DCM; (e) 2D panel of internal water phase-DCM; (f) 2D panel of internal water phase-PLGA; and (g)–(i) 3D response surface plots.

The proportion of DCM and the concentration of PLGA and RWO were the three factors that had an obvious influence on the quality of microcapsules. The Box–Behnken software was used to design response surface experiment (Table 6), and the encapsulation rate was taken as the evaluation index. The predicted results of response surface showed that when the oil phase composition (DCM: EAC) was 40%, PLGA was 160 mg/mL and RWO was 10%, and the EE% was up to 97.83% (Figure 3).

### 3.4. Validation of Optimum Preparation

**Figure 4:**
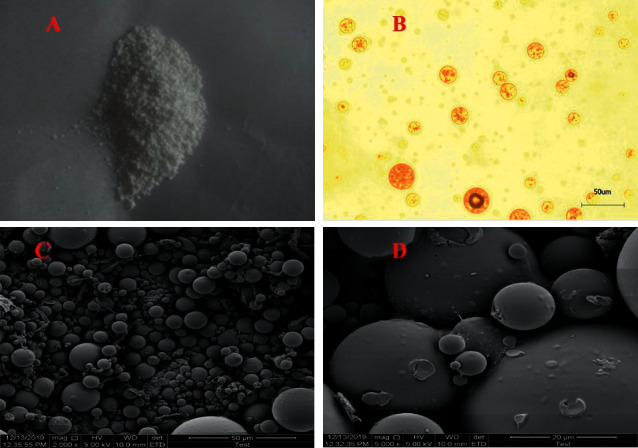
The morphology of ET-PLGA microcapsules. (a) Freeze-dried powder of ET-PLGA microcapsules; (b) the morphology of ET-PLGA microcapsules under an upright microscope; and (c) the morphology of ET-PLGA microcapsules under a scanning electron microscope.

According to the results of single-factor and response surface experiments, the optimum parameters were determined as follows: 40% DCM, 160 mg/mL PLGA, 10% RWO, 4% PVA, and emulsifying time 10 min. Based on the optimized results, 3 batches of ET-PLGA microcapsules were prepared in parallel, and their EE, DL, particle size, and morphology were detected in turn. The results demonstrated that the average encapsulation rate was 96.6% (RSD, 0.46%) and the average DL was 4.58% (RSD, 0.98%). Under the microscope and scanning electron microscope, the surface of ET-PLGA microcapsules was smooth without adhesion (Figure 4). The particle size was 27.51 *μ*m (RSD, 1.2%), and the polydispersity index (PDI) was below 0.3, indicating that the distribution of the particles was narrow. After measuring zeta potential, it was found that the average potential of the particle was below −38 mV, indicating that its property was relatively stable (Table 7).

### 3.5. Healing Effect on ABD

**Figure 5:**
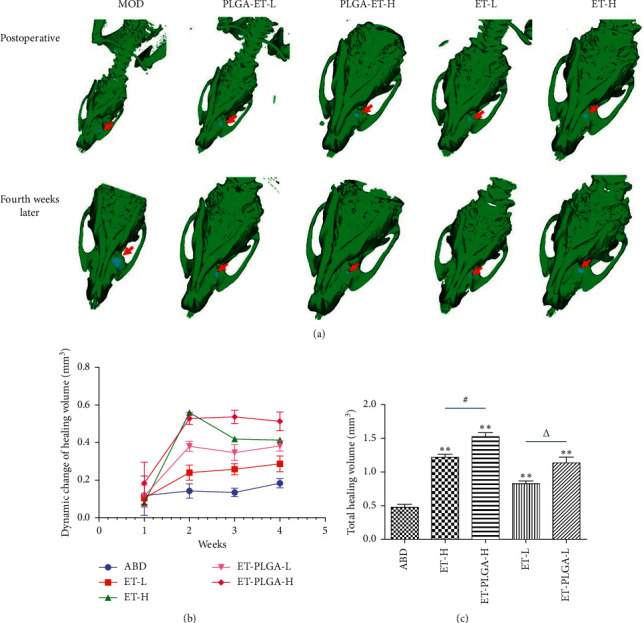
The effect of ET-PLGA microcapsules on alveolar bone defects by CB-CT (*n* = 5). (a) CB-CT images of ABD in rats at different time periods; (b) dynamic changes of healing volume (mm^3^) of ET-PLGA microcapsules in ABD rats; and (c) the total healing volume (mm^3^) of ABD in each group. ^*∗∗*^, compared with the ABD model group, *P* < 0.01; #, compared with the ET-H group, *P* < 0.05; and ∆, compared with the ET-L group, *P* < 0.05.

After administration, the healing volume was calculated once a week for 4 consecutive times. When measuring the healing volume (HV) of bone defect weekly, the calculation method was to subtract the volume of defect (VD) in the previous week from the VD in the current week, such as HV_*n*_ = VD_*n*−1_ − VD_*n*_, *n* = 1, 2, 3, 4. The total healing volume (THV) was calculated as THV = HV_1_ + HV_2_ + HV_3_ + HV_4_. The effects of ET and ET-PLGA microcapsules on the healing of ABD were compared by total healing volume (Figure 5). Compared with the ABD group (0.478 ± 0.100 mm^3^), the total healing volume in all treatment groups was significantly increased (*P* < 0.01). Compared with the ET-L group (0.828 ± 0.075 mm^3^), the total healing volume of ET-PLGA-L (1.133 ± 0.175 mm^3^) was enhanced significantly (*P* < 0.05). Compared with the ET-H group (1.223 ± 0.083 mm^3^), the total healing volume of ET-PLGA-H (1.528 ± 0.113 mm^3^) was also significantly improved (*P* < 0.05). Additionally, there was no abnormal infection, ulceration, or death in the ET-PLGA microcapsule group during the whole experiment.

## 4. Discussion

Compared with the traditional PLGA microspheres, the preparation of PLGA microcapsules with water-phase nuclei has higher technical difficulties [25]. PLGA microspheres have shortcomings in drug loading, encapsulation efficiency, and drug release, especially for water-soluble drugs and hydrophilic macromolecules [26–28]. In this paper, ET-PLGA microcapsules were prepared by double emulsion—solvent evaporation (W_1_/O/W_2_). PLGA polymers were dissolved in a mixture of DCM and EAC (volatile), and then, ET aqueous solution was slowly dripped into it to form an internal aqueous phase, thus forming a W_1_/O colostrum. Then, the W_1_/O colostrum was slowly dripped into the aqueous solution containing 3% PVA and 3% NaCl to form the W_1_/O/W_2_ composite emulsion. Finally, upon DCM and EAC evaporation from the internal oil phase droplets, the solubility of PLGA polymers decreased gradually leading to phase separation and migration to the interface surrounding water droplets.

It was found that the amount of PLGA had a significant impact on the particle size of microcapsules [29]. With the increase of PLGA concentration, the size of microcapsules decreased from several microns to several hundred nanometers. PLGA not only acted as a drug carrier but also played the role of an emulsifier when the internal water phase was encapsulated into the oil phase [30–32]. It could be inferred that the stability of the colostrum was one of the key factors affecting the EE of microcapsules. In addition, due to the presence of PVA in the external water phase, it had the function of a surfactant. When the colostrum was added into the external aqueous phase to prepare the compound emulsion, the outer wall of this prepared microcapsule was compact and uniform [33–35].

Determination of ET and calculation of bone defect volume were the other two technical difficulties in this paper. Fortunately, an ion chromatography method was successfully established, which completed the analysis of a single sample within 10 min with high precision. This powerful analysis method provided important technical support for the optimization of microcapsules. On the other hand, evaluation of the enhancement effect of microcapsules on ABD repair lasted for 1 months and the scanning by CB-CT was needed every week. CB-CT, as a modern noninvasive imaging technique, was widely used in radiology, orthopedics, dentistry, and image-guided radiation therapy [36, 37]. Compared with micro-CT and high-resolution CT, CB-CT recovered imaging parameters accurately, leading to superior image quality [38]. CB-CT provided strong technical support for the evaluation of synergism of ET-PLGA microcapsules in this study.

## 5. Conclusions

An improved double emulsion/solvent evaporation approach was employed to prepare ET-PLGA microcapsules successfully. These microcapsules were spherical in shape and fairly monodisperse in size with a mean particle size of 27.51 *μ*m (PDI = 0.3). The results demonstrated that the average encapsulation rate was 96.6% and the average DL was 4.58%. ET-PLGA microcapsules significantly enhanced the effect of ET on alveolar bone defects. This study provided an important research foundation for the local use of bisphosphonates. More importantly, the implantable PLGA microcapsule system constructed in this study was especially suitable for water-soluble active ingredients (polysaccharides or peptides) with low oral bioavailability in natural medicine.

## Figures and Tables

**Table 1:** The effect of oil phase composition on the preparation of PLGA microcapsules.

Proportion of DCM (%)	EE (%)	Particle size (nm)
30	87.11	12085.61
45	84.94	21872.26
60	84.59	27462.43
75	80.92	27174.97

**Table 2:** Effect of PLGA concentration on the preparation of PLGA microcapsules.

PLGA concentration (mg/mL)	EE (%)	Particle size (nm)
80	74.94	7784.28
120	87.55	9500.88
160	84.74	25136.59
200	92.94	25732.73

**Table 3:** Effect of PVA concentration on the preparation of PLGA microcapsules.

PVA concentration (%)	EE (%)	Particle size (nm)
1	90.88	56577.15
2	89.67	95270.58
3	82.65	19958.97
4	98.79	11593.56

**Table 4:** Effect of emulsification time on the preparation of PLGA microcapsules.

Emulsification time (min)	EE (%)	Particle size (nm)
5	83.53	18711.51
10	86.88	82029.78
15	84.94	13068.48
20	84.94	18896.24

**Table 5:** Effect of volume ratio of the internal water phase to oil phase on the preparation of PLGA microcapsules.

Proportion of the internal water phase (%)	EE (%)	Particle size (nm)
5	93.47	38459.89
10	78.37	19318.54
15	85.03	20178.52
20	78.23	20780.43

**Table 6:** Response surface experiment design.

Std	Run	A	B	C	EE%
2	1	1	−1	0	96.88
7	2	−1	0	1	93.49
1	3	−1	−1	0	94.90
16	4	0	0	0	95.78
15	5	0	0	0	96.88
10	6	0	1	−1	96.78
6	7	1	0	−1	96.88
4	8	1	1	0	96.78
11	9	0	−1	1	96.94
5	10	−1	0	−1	96.95
3	11	−1	1	0	96.93
14	12	0	0	0	95.28
9	13	0	−1	−1	96.97
17	14	0	0	0	96.92
8	15	1	0	1	96.97
13	16	0	0	0	96.91
12	17	0	1	1	96.94

“−1”: 20% DCM, PLGA 160 mg/mL, 5% proportion of the internal water phase; “−0”: 30% DCM, PLGA 180 mg/mL, 7.5% proportion of the internal water phase; “1”: 40% DCM, PLGA 200 mg/mL, 10% proportion of the internal water phase.

**Table 7:** Particle size and potential of ET-PLGA microcapsules.

Batch	Particle size (nm)	Polydispersity	Zeta potential (mV)	EE%	DL%
1	2740	0.26	−38.29	96.65	4.64
2	2717	0.30	−38.63	97.12	4.57
3	2795	0.29	−38.33	96.03	4.53
Average value	2751	0.28	−38.42	96.60	4.58

## Data Availability

The data used to support the findings of this study are available from the corresponding author upon request.

## Supplementary Materials

Supplementary Materials Table S1. The precision of ET by ion chromatography. Table S2. The stability of ET by ion chromatography. Table S3. The repeatability of ET by ion chromatography. Table S4. The Recovery test of ET by ion chromatography.
