# Supplementary material for: Application of Implantable Polylactic-Co-Glycolic Acid Microcapsule in Repairing Alveolar Bone Defects
Source: Evid Based Complement Alternat Med. 2021 Jul 27;2021:5580785. doi: 10.1155/2021/5580785 (PMC8337143; doi:10.1155/2021/5580785)
Supplement: Supplementary Materials — Table S1. The precision of ET by ion chromatography. Table S2. The stability of ET by ion chromatography. Table S3. The repeatability of ET by ion chromatography. Table S4. The Recovery test of ET by ion chromatography. [file 5580785.f1.docx]

**Supplementary materials**

**Precision**

Table S1. The precision of ET by ion chromatography

| No. | Area | Average | SD | RSD |
| --- | --- | --- | --- | --- |
| 1 | 2.011 |  |  |  |
| 2 | 1.986 |  |  |  |
| 3 | 1.976 | 1.949 | 0.046 | 2.34% |
| 4 | 1.929 |  |  |  |
| 5 | 1.910 |  |  |  |
| 6 | 1.881 |  |  |  |

**Stability**

Table S2 The stability of ET by ion chromatography

| Time/h | Area | Average | SD | RSD |
| --- | --- | --- | --- | --- |
| 0 | 25.88 |  |  |  |
| 2 | 25.83 |  |  |  |
| 4 | 25.77 | 25.77 | 0.068 | 0.26% |
| 8 | 25.69 |  |  |  |
| 16 | 25.76 |  |  |  |
| 24 | 25.70 |  |  |  |

**Repeatability**

Table S3 The repeatability of ET by ion chromatography

| No. | Area | Average | SD | RSD |
| --- | --- | --- | --- | --- |
| 1 | 21.72 |  |  |  |
| 2 | 21.79 |  |  |  |
| 3 | 21.86 | 21.86 | 0.080 | 0.37% |
| 4 | 21.89 |  |  |  |
| 5 | 21.94 |  |  |  |
| 6 | 21.95 |  |  |  |

**The recovery**

Table S4 The Recovery test of ET by ion chromatography

| Amount added /μg | Measured quantity /μg | Recovery | Average  recovery | RSD |
| --- | --- | --- | --- | --- |
| 160 | 334.24 | 96.2% |  |  |
| 160 | 338.08 | 98.6% |  |  |
| 160 | 344.32 | 102.5% |  |  |
| 320 | 498.08 | 99.3% |  |  |
| 320 | 499.68 | 99.8% | 99.6% | 1.81% |
| 320 | 494.88 | 98.3% |  |  |
| 640 | 817.12 | 99.5% |  |  |
| 640 | 833.76 | 102.1% |  |  |
| 640 | 819.04 | 99.8% |  |  |
